# Parasitized Natural Killer cells do not facilitate the spread of *Toxoplasma gondii* to the brain

**DOI:** 10.1111/pim.12522

**Published:** 2018-03-25

**Authors:** L. Petit‐Jentreau, C. Glover, J. L. Coombes

**Affiliations:** ^1^ Department of Infection Biology Institute of Infection and Global Health University of Liverpool Liverpool UK

**Keywords:** integrins, NK cells, *Toxoplasma gondii*

## Abstract

*Toxoplasma gondii* is a protozoan parasite capable of invading immune cells and co‐opting their migratory pathways to disseminate through the host. Natural Killer (NK) cells can be directly invaded by the parasite and this invasion alters NK cell migration, producing a hypermotile phenotype. However, the consequences of this hypermotile phenotype for the dissemination of *T. gondii* to the brain remain unknown. To address this, C57BL6/J mice were infected with freshly egressed tachyzoites (type II *Prugniaud* strain) or with parasitized NK cells. Under both conditions, parasite loads in the brain were comparable, indicating that parasitized NK cells were not able to facilitate spread of *T. gondii* to the brain. Consistent with this, we found no evidence for the recruitment of endogenous NK cells to the brain at early time points post‐infection, nor any changes in the expression of α4β1 integrin, involved in recruitment of NK cells to the brain. We therefore found no evidence for a role for hypermotile NK cells in delivery of parasites to the brain during acute infection with *T. gondii*.

## 1 INTRODUCTION

The apicomplexan parasite, *Toxoplasma gondii* (*T. gondii*), causes major public health problems worldwide in immunocompromised people and the developing foetus.^1^ Following ingestion of the parasite by the host, *T. gondii* is initially found in the intestine, before rapidly spreading to the lymphoid tissues (draining lymph nodes and spleen) and finally crossing the blood‐brain barrier to establish chronic infection in the brain.^2^ As an obligate intracellular pathogen, *T. gondii,* and more particularly the invasive tachyzoite form, is able to survive and replicate in any nucleated cell, including immune cells. Previous studies have demonstrated that the parasite can utilize immune cells as “Trojan Horses” to disseminate throughout the host.^3^ For example, when parasitized dendritic cells (DCs) were administered intraperitoneally to naïve mice, parasite loads in the brain increased more rapidly than in mice given free tachyzoites, with the greatest differences observed at 4 days post‐inoculation.^4^ Similarly, tachyzoites were observed within CD11b+ blood cells which, upon adoptive transfer, could establish infection in the brain.^5^ The authors describe these CD11b+ cells as monocytes, although it is worth noting that other blood leucocytes, including Natural Killer (NK) cells, express CD11b. *T. gondii* may exploit the natural migratory pathways of host cells, or actively manipulate host cell migration to augment spread. In vitro studies have shown that parasitized DC displays rapid cytoskeletal remodelling, induction of a hypermotile phenotype and enhanced transmigration across endothelial monolayers.^4^, ^6^, ^7^ It is therefore reasonable to suggest that *T. gondii* is transported across the blood‐brain barrier within host immune cells. However, more recent studies reveal that free tachyzoites are present in blood, and in the endothelium of brain, suggesting that the motile extracellular form of the parasite may be capable of crossing blood‐brain barrier without assistance of host cells.^8^ Nevertheless, host immune cells may play an important role in the dissemination from the site of infection to the bloodstream, or in the delivery of parasites to the brain vasculature.

NK cells are cytotoxic cells of the innate immune system, which are mainly involved in the recognition and destruction of virus‐infected cells or tumour cells.^9^ NK cells also play an important protective role during parasitic infections such as *T. gondii*.^10^ Together with ILC1, NK cells are considered to be part of the group 1 innate lymphoid cell (ILC) family.^11^ In the murine model, *T. gondii* has traditionally been considered to stimulate NK cell responses, and depletion of NK cells results in a higher parasite burden at early stages of the infection.^12^, ^13^ This control is mainly due to the capacity of NK cells to secrete high amounts of interferon γ (IFN‐ γ)^14^ which potentiates activation and differentiation of macrophages/monocytes and DCs, leading to enhanced killing of the parasite and supporting activation of the T cell response.^15^, ^16^, ^17^ More recently, the complexity of the ILC family has been better appreciated, and it is possible that some of the protective function attributed to NK cells may actually derive from ILC1 cells.^18^ Despite their protective role in the immune response against *T. gondii*, NK cells can themselves be parasitized via transmission of *T. gondii* from infected DCs.^19^ Consistent with this, small numbers of *T. gondii*‐invaded NK cells are present in the lymph nodes of infected animals.^20^ This invasion alters NK cell migration, producing a hypermotile phenotype, which may be associated with impaired interactions between integrins and their ligands.^20^ However, we do not yet understand whether this hypermotile phenotype allows NK cells to act as Trojan horses in spread of infection. We hypothesized that the hypermotile phenotype of parasitized NK cells allows them to act as Trojan horses in the transport of tachyzoites to the brain, establishing chronic infection. To evaluate this, we adoptively transferred *T. gondii*‐invaded NK cells into naïve mice and determined whether this led to increased parasite loads in brain compared with the transfer of free tachyzoites.

## 2 METHODS

### 2.1 Mice

Female C57BL/6J mice aged from 8 to 12 weeks were purchased from Charles River (Margate, UK). Animals were housed in specific pathogen‐free conditions and maintained under barrier conditions in individually ventilated cages in accredited animal facilities at the University of Liverpool, UK. All animal procedures were performed in accordance with the UK Scientific Procedures Act of 1986. All experimental protocols were approved by the UK Home Office and the University of Liverpool Animal Committee.

### 2.2 NK cell culture

NK cells were isolated from the spleen of female C57BL/6J mice by negative selection using Easy Sep^™^ Mouse NK cell isolation kit (Stem Cell Technologies). Isolated NK cells were cultured in DMEM—10%, foetal bovine serum (FBS)—1%, HEPES—1%, Penicillin/Streptomycin—0.1%, 2‐mercaptoethanol with 1000 IU/mL recombinant murine IL‐2 (Peprotech). Media and cytokines were replenished every 2‐3 days. IL‐2 cultured NK cells were used after 12‐14 days of culture, after approximately 5‐fold expansion.

### 2.3 Parasites and infection

GFP‐expressing type II *T. gondii* (*Prugniaud*) were a kind gift from Eva Frickel's laboratory. Parasites were cultured in Human Foreskin Fibroblast (HFF) cell line in DMEM‐10% FBS. For in vitro infections, free tachyzoites were harvested by repeated passage of HFFs through a blunt‐ended needle and filtration through a 3 μm nitrocellulose membrane (Whatman). All parasites and human cell lines were regularly tested for Mycoplasma contamination and were confirmed to be negative. IL‐2 cultured NK cells were infected with freshly isolated tachyzoites at a MOI 10:1 (parasite per cell) for 4 hours (with an infection rate between 30% and 77%), washed twice and kept in PBS (“parasitized NK cells”). For in vivo infections, mice were injected intraperitoneally (ip) with 200 μL PBS containing either 2 × 10^4^ free tachyzoites or 2 × 10^3^ parasitized NK cells infected with 2 × 10^4^ tachyzoites. Both samples were subjected to identical centrifugation and washing steps to ensure that total number of parasites transferred was equivalent between groups. At 4 or 7 days post‐infection (dpi), mice were killed by cervical dislocation and organs were harvested.

### 2.4 Single‐cell suspension and flow cytometry

Spleens and brains were harvested and washed in PBS. A single‐cell suspension was obtained using 70 μm cell strainers. Erythrocytes were lysed in ammonium‐chloride‐potassium lysis buffer (0.15M NH_4_Cl, 1 mM KHCO_3_, 0.1 mM EDTA, H_2_O). Fc receptors were blocked with an anti‐CD16/CD32 antibody (clone 2.4G2; ThermoFisher Scientific) for 15 minutes at 4°C and cells were stained for 30 minutes at 4°C with antibodies against: CD3 (clone 17A2; eBiosciences), NK1.1 (clone PK136; Biolegend), CD49b (clone HMa2; eBiosciences), CD29 (clone HMb1‐1; eBiosciences), CD49d (clone R1‐2; Biolegend) and associated isotype controls. Cells were fixed in PBS‐4% formaldehyde (ThermoFisher Scientific), washed and analysed with a MACSQuant cytometer (Myltenyi Biotec) and with FlowJo software (Treestar).

### 2.5 DNA extraction and Q‐PCR

For the detection of *T. gondii*, mice were killed by cervical dislocation; spleens and brains were rapidly harvested and were directly snap‐frozen on dry ice. Organs were individually homogenized in ceramic bead tubes (Precellys) and genomic DNA was extracted using the DNeasy Blood & Tissue Kit from Qiagen. Detection of parasite DNA was evaluated by Q‐PCR using the Power up SybrGreen Master mix (ThermoFisher Scientific) with 50 μg gDNA per sample and with primers specific for the *T. gondii* B1 gene (present in all strains): 5′‐AACGGGCGAGTAGCACCTGAGGAG‐3′ and 5′‐TGGGTCTACGTCGATGGCATGACAAC‐3′. Data were normalized to 50 μg DNA from pure egressed GFP‐*Prugniaud* tachyzoites.

### 2.6 Statistical analysis

Data are expressed as means ± SEM and were analysed using Prism 7 software (GraphPad Software Inc.) for one‐way analysis of variance (ANOVA) with Bonferroni's post hoc test. A *P* value < .05 was considered significant and are indicated with asterisks. ns is not significant.

## 3 RESULTS

### 3.1 NK cells do not accumulate in the brain early after *Toxoplasma gondii* infection

**Figure 1:**
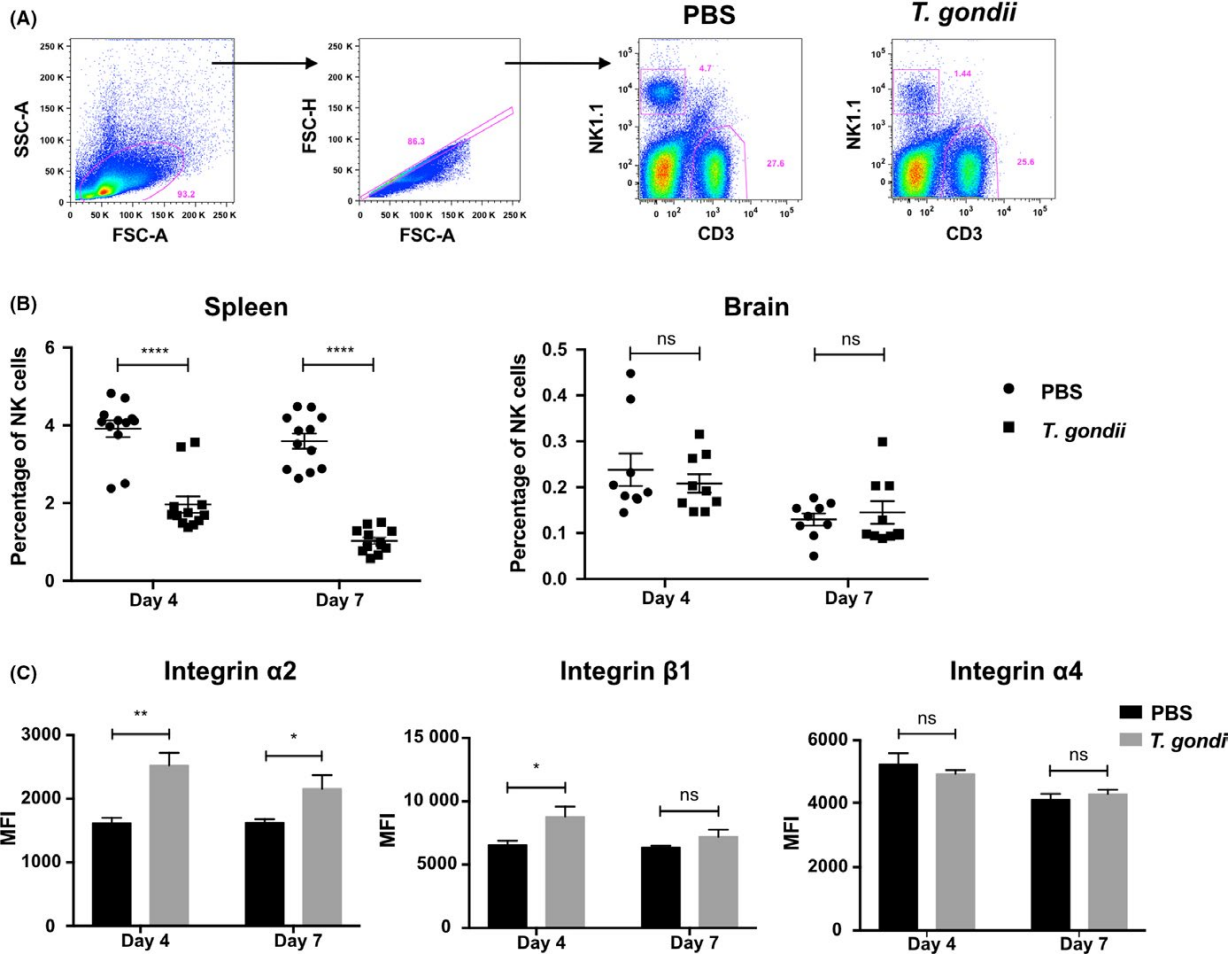
NK cells do not accumulate in the brain early after *Toxoplasma gondii* infection. (A) Flow cytometric analysis showing the percentage of NK cells and T cells in the spleen 4 d post‐injection/infection (dpi). C57BL/6 mice were injected intraperitoneally with PBS or 2.10^4^ free tachyzoites (*T. gondii*). Plots show the gating strategy from one representative experiment at 4 dpi with all events, single cells and NK cells (NK1.1^+^ CD3^‐^) or T cells (NK1.1^‐^ CD3^+^). (B) Flow cytometric analysis showing the percentage of NK cells in the spleen (left panel) and the brain (right panel) 4 or 7 dpi with PBS (black circles) or 2.10^4^ free tachyzoites (black squares) (mean ± SEM, n = 6, pooled from two independent experiments). (C) Flow cytometric analysis showing the Median Fluorescence Intensity (MFI) of integrin α2 (left panel), integrin β1 (middle panel) and integrin α4 (right panel) in the spleen of naïve animals (black bars) or infected animals (grey bars) 4 or 7 dpi (mean ± SEM, n = 6, pooled from two independent experiments). One‐way analysis of variance was performed with a Bonferroni post hoc analysis for multisample testing. *P < .05, **P < .01 and ***P < .001. Abbreviation: ns, not significant

If NK cells play a role in initial transport of *T. gondii* to the brain, we might expect to see recruitment of NK cells to the brain or associated vasculature early after infection. To address this, female C57BL6/J mice were infected with freshly egressed tachyzoites of the type II *Prugniaud* strain or PBS as a control. At 4 and 7 days post‐infection (dpi), we determined the percentage of NK cells in a lymphoid tissue (where NK cells become parasitized) and in the brain (a preferential site for the establishment of chronic *T. gondii* infection).^2^, ^21^, ^22^ These time points were selected as they coincide with the earliest infiltration of *T. gondii* into the brain, when we might expect immune cell‐mediated trafficking to be important.^4^ The percentage of NK cells in the spleen decreased from 3.75% in noninfected animals to 1.96% in infected animals after 4 dpi and from 3.6% to 1% after 7 dpi (Figure 1A,B), suggesting that NK cells may have migrated away. This is consistent with other *T. gondii* infection models^12^, ^23^ and with the idea that inflammation induces mobilization of NK cells from storage depots in the spleen to the blood and inflamed tissue.^24^ However, after 4 or 7 dpi, the percentage of NK cells was similar in the brain of noninfected and infected animals (Figure 1B). These data demonstrate that there was no increase in the presence of NK cells in the brain and associated vasculature early after infection.

### 3.2 Expression of α2β1, but not α4β1 is modulated following infection

Although we found no evidence for the infiltration of NK cells into the brain and associated vasculature early after infection, the decreased percentage of NK cells in the spleen of infected animals suggested that NK cells migrate differently after *T. gondii* infection. Integrins play an important role in cell adhesion and migration.^25^ α2β1 integrin has been shown to drive accumulation and retention of NK cells in the subcapsular region of lymph nodes of *T. gondii*‐infected mice,^12^ while α4β1 integrin regulates recruitment to the central nervous system (CNS).^26^, ^27^ To address whether the decrease in splenic NK cells was accompanied by changes in integrin expression, we evaluated the expression of the α2, α4 and β1 integrin subunits in NK cells in the spleen 4 or 7 dpi. Contrary to expectations, at day 4 pi, α2 and β1 expression significantly increased in the NK cells in the spleen of infected animals compared with uninfected controls (Figure 1C). α2 expression on NK cells remained significantly higher in *T. gondii*‐infected mice compared with noninfected animals at d7 dpi (Figure 1C). However, α4 expression on NK cells was similar between infected animals and noninfected animals (Figure 1C), consistent with the lack of NK cell accumulation observed in the brains of infected animals (Figure 1B).

### 3.3 Integrin expression is unchanged in parasitized NK cells

**Figure 2:**
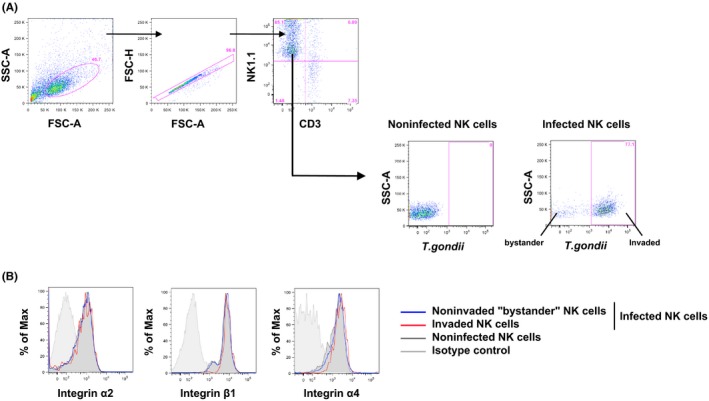
Integrin expression is not modulated in parasitized NK cells. IL‐2 cultured NK cells infected with type II *Prugniaud* tachyzoites expressing the GFP with MOI 10:1 for 4 h. (A) Flow cytometric analysis of IL‐2 cultured NK after 4 h of infection. Plots show gating of all events (SSC‐A/FSC‐A), single cells (FSC‐H/FSC‐A) and NK cells (NK1.1^+^ CD3^‐^). Percentage of infected NK cells is then determined by gating on parasite fluorescence (GFP‐positive population). This is a representative experiment from more than 3 different experiments. (B) Flow cytometric analysis of integrin α2 (left panel), integrin β1 (middle panel) and integrin α4 (right panel) expression in IL‐2 cultured NK cells after 4 h of infection. Blue lines correspond to the “bystander” NK cells (GFP ^‐^ in infected samples), red lines to the invaded NK cells (GFP ^+^ in infected samples) and black lines to the noninfected NK cells (naïve population). Grey lines show the corresponding isotype controls. This is a representative data from more than 3 different experiments

We have previously shown that parasitized NK cells migrate differently to bystander NK cells in lymphoid tissues and that integrin clustering is impaired in these cells.^20^ Therefore, although the changes we observed in integrin expression in the bulk NK cell population were not consistent with migration to the brain (Figure 1B,C), we wanted to determine if integrin expression was differentially regulated on directly parasitized NK cells compared with population as a whole. However*, in vivo*, only a very small proportion of NK cells are directly invaded by the parasite,^20^ making this difficult to assess (Figure 1B). To circumvent this, cultured NK cells isolated from the spleen of naïve C57BL6/J mice were infected with the type II *Prugniaud* strain expressing GFP. After 4 hours of infection, we found that 30%‐77% of NK cells were directly invaded by the parasite as determined by GFP in NK cells (Figure 2A). This range is in accordance with recently published data.^28^ We then assessed the expression of the three integrin subunits α2, α4 and β1 after direct invasion of NK cells with the parasite. After infection, α2, α4 and β1 expressions were similar between the invaded NK cells and “bystander” NK cells (Figure 2B). There were also no differences between the naïve NK population and the invaded NK cells (Figure 2B), suggesting that α2β1 and α4β1 integrin expressions on NK cells were unchanged after direct invasion with the parasite.

### 3.4 *Toxoplasma gondii* does not spread more efficiently to the brain when contained within NK cells

**Figure 3:**
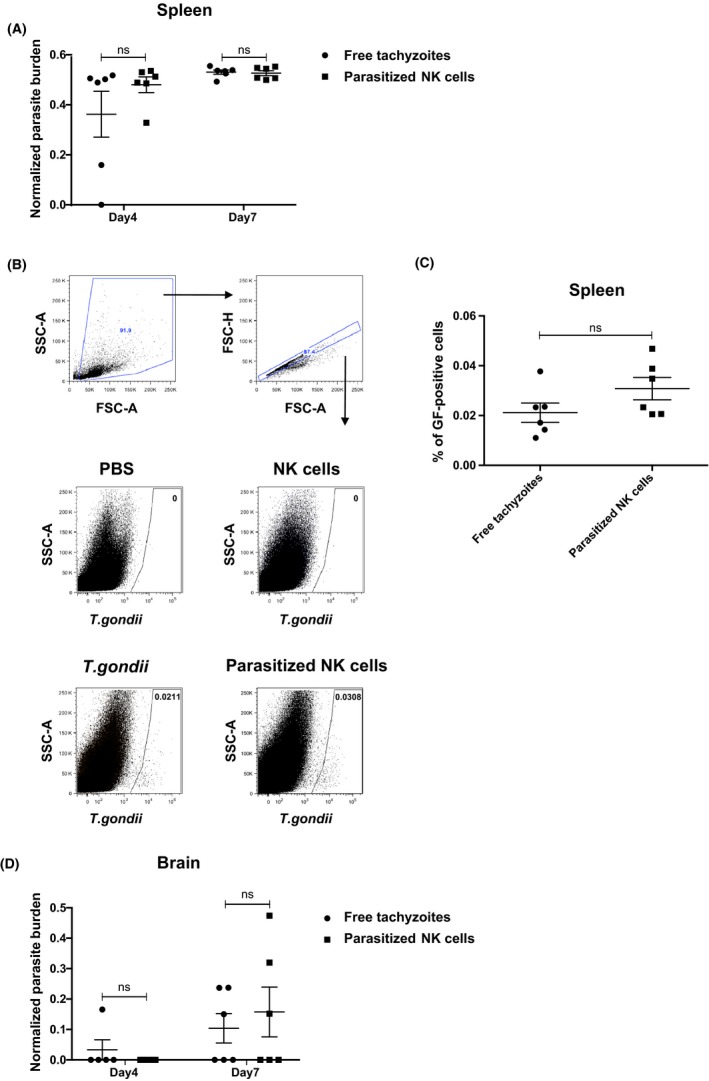
*Toxoplasma gondii* does not spread more efficiently to the brain when contained within NK cells. (A) Parasite burden was quantified and normalized by Q‐PCR in the spleen of *T. gondii* (black circles)‐ or parasitized NK cell (black squares)‐injected groups after 4 or 7 dpi. Normalization was performed with DNA of pure egress tachyzoites. (mean ± SEM, n = 6, pooled from two independent experiments). (B) Flow cytometric analysis showing the percentage of infected cells 7 dpi in the spleen of animals injected with 2.10^4^ free tachyzoites (*T. gondii*) ip or 2.10^3^ parasitized NK cells ip Groups injected with PBS ip or 2.10^3^ naïve NK cells ip were shown as controls. Plots show gating of all events, single cells and infected cells based on the parasite fluorescence. (C) C57BL6/J mice were intraperitoneally injected with *T. gondii* (black circles) or parasitized NK cells (black squares) and the percentage of infected cells were analysed in the spleen 7 dpi. (mean ± SEM, n = 6, pooled from two independent experiments). (D) Parasite burden was quantified and normalized by Q‐PCR in the brain of *T. gondii* (black circles)‐ or parasitized NK cell (black squares)‐injected groups after 4 or 7 dpi (mean ± SEM, n = 6, pooled from two independent experiments). One‐way analysis of variance was performed with a Bonferroni post hoc analysis for multisample testing. *P < .05, **P < .01 and ***P < .001. Abbreviation: ns, not significant

Adoptive transfer of parasitized DCs or CD11b^+^ cells can transport the parasite into the host when they contained the parasite,^4^, ^29^ allowing the access of tachyzoites into the brain.^5^, ^30^ To determine if NK cells can also play a role in spread of *T. gondii* to the brain, we compared parasite loads in the brains of mice infected with free tachyzoites or with in vitro parasitized NK cells. Splenic NK cells were selected as, upon adoptive transfer, they have been shown to recirculate through all organs ordinarily containing NK cells.^31^ Previous studies showed that tachyzoites are detected in lymphoid tissues early after infection (between 2 and 6 days),^4^, ^12^, ^32^, ^33^ while parasites were detected in the brains of infected animals by 7 dpi.^4^, ^8^, ^30^, ^34^ We therefore decided to evaluate the parasite burden in the spleen and the brain of infected animals by PCR at 4 and 7 dpi. Mice were administered either free tachyzoites, or *T. gondii*‐infected NK cells (pre‐injection analysis shown in Figure 2A) and checked for their weight loss at different time post‐infection (Figure S1). At 4 dpi, parasite DNA was detectable in the spleen of all mice infected with parasitized NK cells, and 5 of 6 mice infected with free tachyzoites (Figure 3A). At 7 dpi, parasite DNA levels in the spleen were comparable between mice given free tachyzoites, and mice given parasitized NK cells (Figure 3A). Flow cytometric analysis of percentage of GFP‐positive cells (based on the parasite GFP fluorescence) in the spleen at day 7 post‐infection confirmed these findings (Figure 3B,C). We then assessed if parasites can spread more efficiently to the brain when contained within NK cells. At 4 dpi, parasites were just beginning to arrive in the brain, being detectable in the brain of one mouse infected with free tachyzoites, but not in the brains of mice injected with parasitized NK cells (Figure 3D). However, by 7 dpi, parasites were detected in the brains of 3 of 6 mice infected with either free tachyzoites or parasitized NK cells but no significant differences were observed in the parasite burden between the two groups (Figure 3D). Moreover, the percentage of NK cells was similar in the spleens and the brains between the two groups at 4 and 7 dpi (Figure S2). Overall, these findings demonstrate that *T. gondii* does not spread more efficiently to the brain when it has been contained within NK cells.

## 4 DISCUSSION

Despite their protective role during parasitic infections, recent evidence indicates that NK cells can be directly invaded by *T. gondii*.^19^, ^20^ *T. gondii*‐invaded NK cell are not efficiently targeted for destruction by other NK cells and are hypermotile.^19^, ^20^ This may allow the parasite to use NK cells to protect themselves from immune mediated destruction, while transiting successfully from the site of infection to muscles or brain. However, the importance of this pathway remains unknown. Here, we used a model of infection with free tachyzoites or parasitized NK cells to elucidate the consequences of NK cell invasion in the transport of *T. gondii* throughout the host. Our data show that infection with parasitized NK cells does not increase infection levels in brain relative to infection with parasites alone. We therefore find no evidence that NK cells play a significant role in the establishment of chronic infection by *T. gondii*.

The inability of parasitized NK cells to potentiate spread of parasites to the brain is somewhat surprising given the previous literature supporting roles for other immune cell populations in the spread of infection.^35^ For example, adoptive transfer of *T. gondii*‐infected DCs into the peritoneal cavity resulted in higher infection loads in the brain at early time points post‐infection, when compared with infection with free parasites.^4^, ^5^, ^7^ Furthermore, blood CD11b+ cells (which may include NK cells) were able to shuttle tachyzoites across the blood‐brain barrier.^5^, ^30^ However, in agreement with our findings, transfer of a mixed lymphocyte population (predominantly B cells) did not augment parasite dissemination relative to free tachyzoite controls.^36^ Our findings are also in agreement with more recent data demonstrating that free tachyzoites are able to cross the blood‐brain barrier without the assistance of host immune cells.^8^ While this latter study did not exclude the idea that immune cells may facilitate invasion of endothelial cells by bringing parasites in close contact with endothelial cells, our own analysis showed no increase in the presence of NK cells in either the brain or associated vasculature following infection.

Using two‐photon microscopy of living tissue from *T. gondii*‐infected mice, we have previously shown that *T. gondii*‐infected NK cells showed faster, more directed and more persistent migratory behaviour when compared with uninfected “bystander” NK cells.^20^ Contrary to DCs, the hypermotile phenotype in NK cells is observed only in tissues, and not in simplified in vitro assays.^36^ Therefore, while NK cell hypermotility clearly occurs *in vivo* during natural infections, it has been challenging to determine the mechanism underlying this hypermotile phenotype. Following infection, NK cells formed α2‐integrin‐dependent contacts with collagen fibres, which reduced NK cell motility and played an important role in the retention of NK cells in foci of infection.^12^ Consistent with this, we observe an increase in cell surface α2 on the total splenic NK cell population following infection. In contrast to the bulk NK cell population, parasitized NK cells become hypermotile, and we might therefore expect a decrease in α2 expression.^20^ However, our analysis of in vitro parasitized NK cells suggests that this is not the case. Expression of the integrin α4β1, involved in migration of NK cells to the CNS, was also unchanged in in vitro parasitized NK cells. This is in direct contrast with an earlier study, which reported a down modulation of α4 integrin on infected macrophages.^37^ Instead, the parasite may modulate integrin activity or signalling in host cells. Consistent with this, we and others have described impaired clustering of integrins in parasitized immune cells.^2^, ^20^ Determining how the parasite achieves this may give clues to the general principles governing NK cell migration, allowing for therapeutic manipulation in other disease settings.

If NK cells are not involved in shuttling parasite to brain, does parasite derive any benefit for invading NK cells? A recent study showed that direct invasion with *T. gondii* could also impaired NK cell effector function.^28^ Consistent with this, we have previously found impaired clustering of the integrin LFA‐1, involved in NK cell cytotoxicity, in parasitized NK cells.^20^ We can speculate that invasion of NK cells by the parasite leads to impaired formation of lytic contacts. However, in physiological models of *T. gondii* infection, the percentage of invaded NK cells is very low suggesting that impaired capacities of parasitized NK cells are negligible for the outcome of the infection.

In conclusion, we find no evidence for a role for NK cells in early shuttling of *T. gondii* to the host brain. Nevertheless, it will be of interest to determine the molecular mechanisms underlying parasite‐induced changes in NK cell migration so that we may modulate NK cell homing in other disease settings.

## CONFLICT OF INTEREST DISCLOSURE

The authors declare no competing financial interests.

## AUTHORSHIP

LP‐J and CG performed the experiments. LP‐J analysed the data. LP‐J and JLC conceived, designed the study and wrote the manuscript.
