# Supplementary figures and images for: Parasitized Natural Killer cells do not facilitate the spread of Toxoplasma gondii to the brain
Source: Parasite Immunol. 2018 Mar 25;40(4):e12522. doi: 10.1111/pim.12522 (PMC5901034; doi:10.1111/pim.12522)

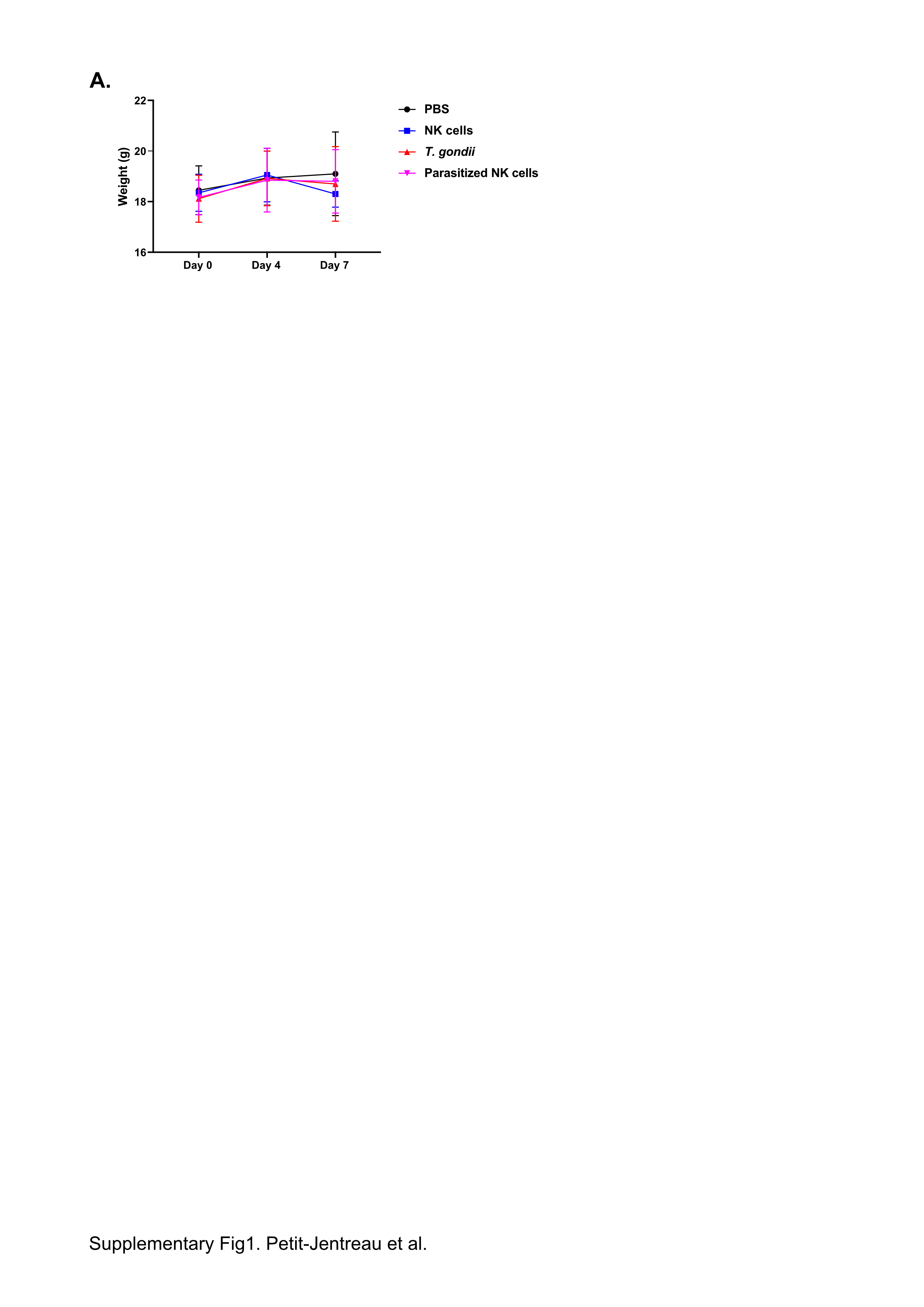

Supplement: Supplementary file 1 [file PIM-40-na-s001.jpg]

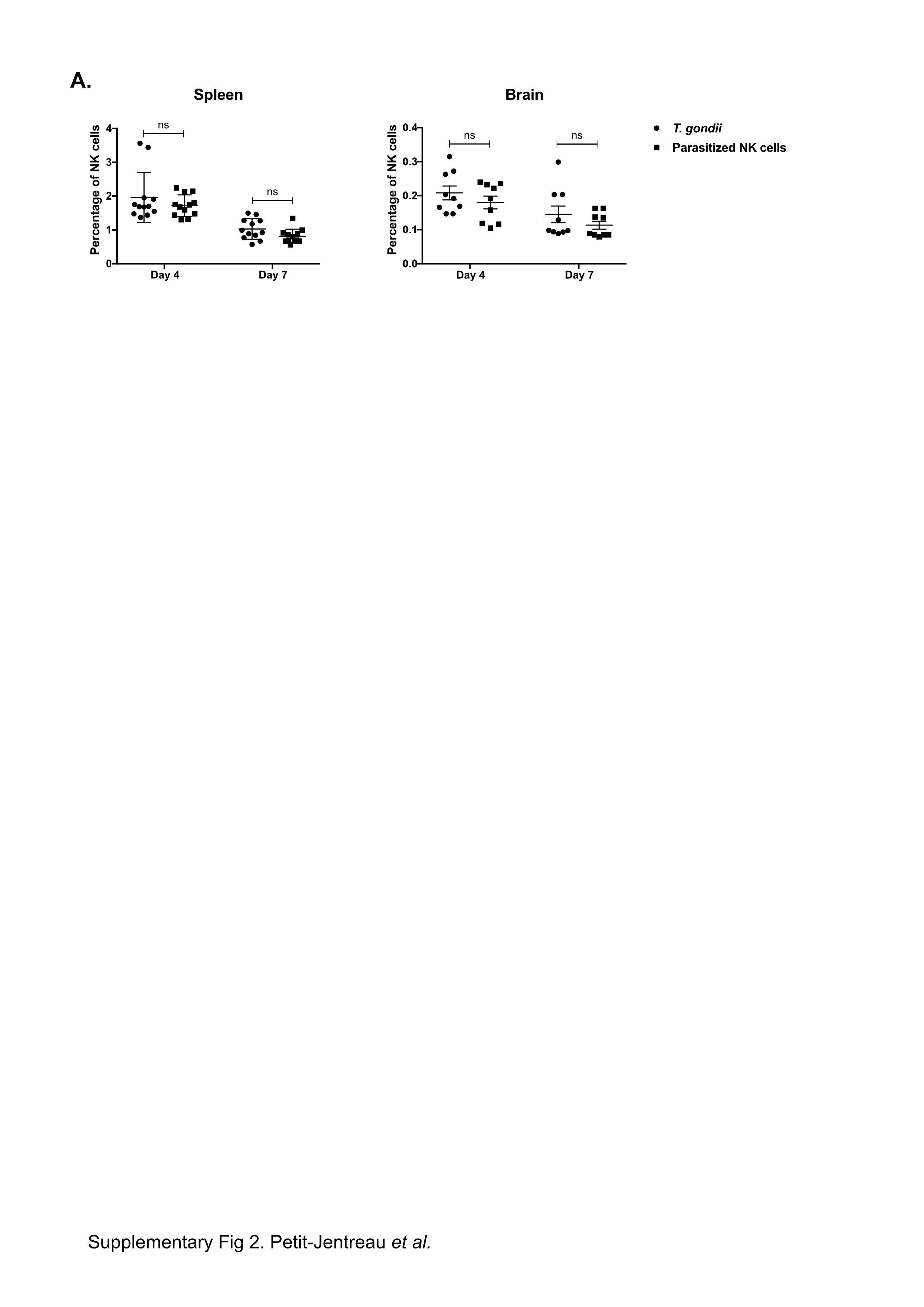

Supplement: Supplementary file 2 [file PIM-40-na-s002.jpg]
